# Biological rationale and potential clinical use of gabapentin and pregabalin in bipolar disorder, insomnia and anxiety: protocol for a systematic review and meta-analysis

**DOI:** 10.1136/bmjopen-2016-013433

**Published:** 2017-03-27

**Authors:** Kerensa T Houghton, Alexandra Forrest, Amine Awad, Lauren Z Atkinson, Sarah Stockton, Paul J Harrison, John R Geddes, Andrea Cipriani

**Affiliations:** 1 Department of Psychiatry, University of Oxford, Warneford Hospital, Oxford OX3 7JX, UK; 2 Oxford Health NHS Foundation Trust, Warneford Hospital, Oxford OX3 7JX, UK

**Keywords:** MENTAL HEALTH, Bipolar disorder, Insomnia, Systematic review, Meta-analysis

## Abstract

**Introduction:**

Gabapentin has been extensively prescribed off-label for psychiatric indications, with little established evidence of efficacy. Gabapentin and pregabalin, a very similar drug with the same mechanism of action, bind to a subunit of voltage-dependent calcium channels which are implicated in the aetiopathogenesis of bipolar disorder, anxiety and insomnia. This systematic review and meta-analysis aims to collect and critically appraise all the available evidence about the efficacy and tolerability of gabapentin and pregabalin in the treatment of bipolar disorder, insomnia and anxiety.

**Methods and analysis:**

We will include all randomised controlled trials (RCTs) reported as double-blind and comparing gabapentin or pregabalin with placebo or any other active pharmacological treatment (any preparation, dose, frequency, route of delivery or setting) in patients with bipolar disorder, anxiety or insomnia. For consideration of adverse effects (tolerability), single-blind or open-label RCTs and non-randomised evidence will also be summarised. The main outcomes will be efficacy (measured as dichotomous and continuous outcome) and acceptability (proportion of patients who dropped out of the allocated treatment). Published and unpublished studies will be sought through relevant database searches, trial registries and websites; all reference selection and data extraction will be conducted by at least 2 independent reviewers. We will conduct a random-effects meta-analysis to synthesise all evidence for each outcome. Heterogeneity between studies will be investigated by the I^2^ statistic. Data from included studies will be entered into a funnel plot for investigation of small-study effects. No subgroup analysis will be undertaken, but we will carry out sensitivity analyses about combination treatment, psychiatric comorbidity, use of rescue medications and fixed versus random-effects model.

**Ethics and dissemination:**

This review does not require ethical approval. This protocol has been registered on PROSPERO (CRD42016041802). The results of the systematic review will be disseminated via publication in a peer-reviewed journal.

**Strengths and limitations of this study**

- Gabapentin and pregabalin have been extensively prescribed off-label for psychiatric indications, including bipolar disorder, sleep and anxiety, with little established evidence of efficacy. We will conduct a systematic review of all available published and unpublished literature and will carry out a random-effects pairwise meta-analysis to synthesise all available evidence for each outcome, if possible.
- To assess efficacy and acceptability, only double-blind, randomised controlled trials will be included, while, for consideration of adverse effects (tolerability), single-blind or open-label trials and non-randomised evidence will also be summarised.
- Gabapentin and pregabalin are ligands of the α2δ subunit of voltage-dependent calcium channels, which are currently under investigation as a target for novel pharmaceuticals to be used in the management of bipolar disorder.
- The risk of publication bias and the risk of selection bias are high in the psychiatric literature, in particular with gabapentin.
- The limitations of primary studies will be addressed with the Cochrane risk of bias tool and the quality of evidence contributing to pooled estimates will be assessed with the GRADE framework.

## Background

Gabapentin was first licenced by the Food and Drug Administration (FDA) in 1993 as an adjunctive treatment for partial seizures in people aged over 12 years.^1^ It was subsequently discovered to have analgesic properties^2–4^ and licenced by the FDA for use in postherpetic neuralgia in 2004. In the same year, pregabalin, a structural analogue of gabapentin, was licenced for the treatment of neuropathic pain and as an adjunct in the management of epilepsy.^5–7^ Pregabalin has since been licenced to treat generalised anxiety disorder.^8^ Gabapentin has a long history of off-label prescription.^9^ Between 1998 and 2000, a study involving 105 Medicaid patients who were prescribed gabapentin found that 95% of prescriptions had been for off-label indications.^10^ A number of these indications were for psychiatric illness, including bipolar disorder (10% of all prescriptions in the study) and anxiety disorders. Despite the prevalent prescription of gabapentin for psychiatric illness spanning over 20 years, there has been limited investigation into its efficacy for such disorders. Berlin *et al*^11^ conducted a systematic review of the use of gabapentin in psychiatric illness, but did not search unpublished trials. They reported mixed evidence as to the efficacy of gabapentin in bipolar disorder. One randomised controlled trial (RCT) comparing adjunctive use of gabapentin in bipolar mania or depression with placebo found no improvement of symptoms in the gabapentin group.^12^ Another two studies, which compared gabapentin monotherapy in refractory bipolar disorder with placebo and lamotrigine, found gabapentin to be ineffective in treating mania or depression.^13^ ^14^ Additionally, a meta-analysis conducted to compare treatments of acute mania found gabapentin to be less efficacious than placebo.^15^ However, a trial which compared gabapentin with placebo in the maintenance of bipolar disorder concluded that gabapentin may provide some benefit in the long-term treatment of bipolar disorder.^16^ The evidence for gabapentin in anxiety disorder is similarly incomplete. Only one RCT has been completed to compare gabapentin and placebo as adjunctive therapy in social phobia, suggesting that gabapentin is more effective than placebo.^17^ Other trials of gabapentin in anxiety have focused on preoperative anxiety and have mostly found gabapentin to be more effective than placebo.^11^

Given the lack of evidence of efficacy of gabapentin in bipolar disorder and anxiety, it may be difficult to understand the widespread prescription of the drug for these off-licence indications. However, the pharmaceutical company which promoted gabapentin, Pfizer, has been fined in the USA for illegal promotion of gabapentin for unlicenced indications.^18^ It is therefore uncertain whether the historical widespread prescription of gabapentin for psychiatric illness was due to efficacy noted by individual physicians and patients, or due to illegal promotion of the drug for off-label use.

Pregabalin has been found to be effective in the management of generalised anxiety disorder,^19^ and is licenced in the UK for this indication. There is also some evidence, from an open-label observational study, that pregabalin is effective in the adjunctive treatment of acute mania, depression, and maintenance of refractory bipolar disorder.^20^ Gabapentin was originally designed to be structurally similar to the neurotransmitter γ-aminobutyric acid (GABA).^21^ It was intended as an antispasmodic drug which would act on GABA receptors and have GABAergic properties.^22^ In fact, neither gabapentin nor pregabalin bind to GABA receptors. Both drugs are ligands of the α2δ subunit of voltage-dependent calcium channels,^23^ ^24^ and there is evidence that their antiepileptic and analgesic properties are directly related to their calcium channel interaction.^25^ ^26^ Voltage-dependent calcium channels are currently under investigation as a target for novel pharmaceuticals to be used in the management of bipolar disorder, and they have four subunits:

-  α1 subunit, which forms the transmembrane calcium-selective pore;^27^
-  β and α2δ subunits, which are involved in trafficking of the α1 subunit to the cell membrane;^28^
-  γ subunit, which is not involved in trafficking, but appears to influence biophysical properties of associated calcium channels.^29^

Voltage-gated calcium channels are classified into three main groups depending on sequence homology of the α1 subunit:^30^ (1) Ca_v_1, or L-type channels; (2) Ca_v_2, of which there are four subtypes known as P, Q, N and R-type channels; (3) Ca_v_3, also called T-type calcium channels. L-type calcium channels (Ca_v_1) may be further classified into types Ca_v_1.1, Ca_v_1.2, Ca_v_1.3 and Ca_v_1.4. The channel Ca_v_1.2, which is found in skeletal muscle, heart and brain, is coded for by the gene *CACNA1C*. Genome-wide association studies have consistently found an association between a common single-nucleotide polymorphism in the *CACNA1C* gene and bipolar disorder,^31^ ^32^ which appears likely to increase Ca_v_1.2 channel expression and function.^33^ ^34^ Other evidence that calcium channels may be involved in the aetiopathogenesis of bipolar disorder is that agonist-stimulated calcium response, which is a key pathway in intracellular secondary messaging, is enhanced in the platelets of patients with bipolar disorder.^35^ Additionally, mitochondria influence the sequestration of excess intracellular calcium which accumulates as a result of agonist stimulation and magnetic resonance studies have found mitochondrial dysfunction in patients with bipolar disorder.^36^ ^37^

This evidence suggests that calcium channels, particularly Ca_v_1.2 channels, may be a useful target in the design of novel therapies for bipolar disorder. Further, since the genetic mutations associated with bipolar disorder are likely to increase Ca_v_1.2 expression and function, drugs which act as antagonists to the action of this channel may be found to be effective in bipolar disorder. A recent review found no evidence that verapamil, an L-type calcium channel blocker usually prescribed for cardiovascular pathology, was effective in the management of acute mania.^38^ However, the review suggests that this might be due to poor blood–brain barrier permeability of verapamil. There are also concerns about cardiovascular side effects of using L-type calcium channel blockers in bipolar disorder.

Gabapentin and pregabalin cross the blood–brain barrier readily^39^ ^40^ and have limited side-effect profiles.^8^ Gabapentin has also been found to reduce peak high-voltage-activated (Ca_v_1 and Ca_v_2) Ca^2+^ channel currents in rat dorsal root ganglion cultured cells, after incubation with the drug for 3 days.^41^ The same study found that gabapentin reduced cell-surface expression of Ca_v_2 channels in xenopus oocytes, although Hoppa *et al*^42^ found no evidence of reduced Ca_v_2 channel current or expression in rat hippocampal cells under the same conditions of gabapentin administration. The results from the study by Hendrich *et* *al*^41^ suggest that gabapentin (and pregabalin, as it binds to the same site on the α2δ subunit of the calcium channel) may be effective at inhibiting calcium channel function and therefore may be useful in the management of bipolar disorder. The *CACNA1C* gene is also implicated in sleep/wake regulation. Genome-wide association studies have found *CACNA1C* mutations are associated with sleep latency and quality^43^ as well as narcolepsy.^44^ Additionally, *CACNA1C* knockout mice have been shown to have altered REM sleep.^45^ This link between calcium channels and sleep quality suggests that gabapentin and pregabalin may influence sleep patterns. Further evidence that this may be the case is that noradrenergic neurons in the locus coeruleus of the human brain fire at different rates depending on sleep state and are therefore implicated in regulation of sleep/wake states.^46^ Gabapentin and pregabalin have been shown to decrease GABA levels, thereby increasing glutamate and promoting descending noradrenergic inhibition in rat's locus coeruleus.^47^ ^48^ Sleep disturbance is often comorbid with bipolar disorder^49^ and increased firing of noradrenergic neutrons in the locus coeruleus has also been associated with stress, fear and anxiety.^50^ Therefore, the actions of gabapentin and pregabalin in the locus coeruleus may be responsible for their anxiolytic effect. Some of the most effective drugs for acute anxiety are the benzodiazepines, which work by increasing the action of GABA at GABA_A_ receptors.^51^ However, the work of Yoshizumi *et al*^47^ and Suto *et al*^48^ suggest that gabapentin reduces presynaptic GABA release in rat's locus coeruleus. Hellsten *et al*^50^ found different expression of GABA_A_ receptors in rodents compared with humans and suggested that the human locus coeruleus may mediate anxiolytic and sedative effects of benzodiazepines. Despite uncertainty in the neurobiological justification for possible anxiolytic efficacy of gabapentin and pregabalin, the latter is licenced for generalised anxiety disorder and gabapentin has been used to treat anxiety disorders for many years.

In summary, gabapentin has been extensively prescribed off-label for psychiatric indications, including bipolar disorder and anxiety, with little established evidence of efficacy. Gabapentin and pregabalin, a very similar drug with the same mechanism of action, bind to a subunit of voltage-dependent calcium channels. These calcium channels are found in the human brain and are implicated in the aetiopathogenesis of bipolar disorder, anxiety and insomnia. Given the neurobiological argument described above and the common clinical coexistence of bipolar disorder, anxiety and insomnia, this systematic review aims to collect and critically appraise all the available evidence about efficacy and tolerability of gabapentin and pregabalin in the treatment of bipolar disorder, insomnia and anxiety.

## Objectives

- To assess the efficacy of gabapentin and pregabalin in bipolar disorder in:

- attenuating acute manic/mixed episodes;
- attenuating acute depressive episodes;
- preventing relapse of any mood episodes.

- To assess the efficacy of gabapentin and pregabalin in the acute and long-term management of anxiety disorders.
- To assess the efficacy of gabapentin and pregabalin in the acute and long-term management of insomnia.

1. To assess the acceptability and tolerability of gabapentin and pregabalin in comparison with placebo or active treatment when used in the acute or long-term treatment of bipolar disorder, insomnia and anxiety.

## Methods

### Types of studies

To assess efficacy and acceptability, only double-blind, RCTs will be included. For consideration of adverse effects (tolerability), single-blind or open-label RCTs and non-randomised evidence will also be summarised. For trials that have a crossover design, only results from the first period prior to crossover will be considered. Cluster randomised trials will be excluded. In bipolar disorder, acute treatment is the intervention given to patients with an acute manic, depressed or mixed episode (usually 3 weeks in manic and mixed episodes^15^ and 8 weeks for depression);^52^ in contrast, long-term treatment is a treatment which had been instituted specifically or mainly to prevent further episodes of illness (either manic, mixed or depressive).^53^ In this review, we defined long-term treatment for bipolar disorder as treatment with a minimum duration of at least 3 months (>12 weeks).^54^ ^55^ For acute treatment of insomnia, the follow-up is usually <1 month, between 1 and 3 months for subchronic insomnia and more than 3 months for persistent insomnia.^56^ The overall aim of treatment for anxiety is to improve—and ideally achieve complete relief from—symptoms and to prevent their recurrence; following an initial acute phase, treatment usually needs to be continued on a long-term basis, due to the chronic nature of the disease.^57^ For trials about anxiety, we will consider the authors' definition of acute and long-term management as reported in the original study.

### Types of participants

Patients of any age, of both sexes, any ethnicity, based in any clinical setting will be included.

#### Bipolar disorder

Only studies adopting standardised diagnostic criteria from the International Classification of Diseases (ICD) or the Diagnostic and Statistical Manual of Mental Disorders (DSM) to define patients suffering from bipolar disorder will be included. We will exclude studies which define bipolar disorder as scoring above a certain cut-off on a screening questionnaire. All subtypes of bipolar disorder (rapid cycling, type I, type II, not otherwise specified) will be included. No restrictions on clinician setting (eg, primary or secondary care) will be applied. Patients who have a concurrent primary diagnosis of an Axis I disorder or a serious concomitant medical illness will be excluded, with the exception of patients with comorbid anxiety and insomnia who will be analysed separately in a sensitivity analysis.

#### Anxiety

Studies relating to any anxiety disorder defined in the ICD or DSM will be included. These include, but are not limited to, panic disorder, generalised anxiety disorder and phobic anxiety disorders. Additionally, we will include studies which investigate anxiety in a population with no diagnosis of anxiety but who are observed during a period of stress, for example, preoperative anxiety. Patients who have a concurrent primary diagnosis of an Axis I disorder or a serious concomitant medical illness will be excluded, with the exception of patients with comorbid bipolar disorder and insomnia who will be analysed separately in a sensitivity analysis.

#### Insomnia

Any studies relating to sleep or insomnia will be included. This includes studies which assess sleep quality and patterns in normal persons as well as those which consider a population of patients with a defined sleep disorder. Patients who have a concurrent primary diagnosis of an Axis I disorder will be included, but patients with other serious concomitant medical illness will be excluded.

### Types of intervention

#### Experimental interventions

Gabapentin or pregabalin in any dose, frequency, route of delivery or setting will be included. Trials in which gabapentin or pregabalin therapy is ‘added-on’ to pre-existing treatments (eg, lithium) will be included if the pre-existing treatments are evenly distributed in the experimental and comparator intervention arms of the study. Trials which allow rescue medications (eg, short-term use of hypnotics) will be included as long as these medications are equally distributed among the randomised intervention and comparator arms. Sensitivity analyses will then be performed to investigate if cotreatment or rescue medications are responsible for altering the efficacy of gabapentin or pregabalin in treating bipolar disorder, insomnia or anxiety.

#### Comparator interventions

Placebo or any other active pharmacological treatment (any preparation, dose, frequency, route of delivery or setting).

### Types of outcome measures

#### Primary outcomes

- Efficacy of gabapentin or pregabalin in the acute treatment of bipolar disorder, is as follows:
  Number of hospital admissions during the study period;
  Length of hospital admission;
  Changes on validated manic or depressive symptom rating scales from baseline;
  Changes on validated psychotic symptom rating scales from baseline;
  Response to treatment (ie, at least 50% improvement on any validated rating scale);
  Time to cessation of additional treatment for manic/depressive symptoms.
- Efficacy of gabapentin or pregabalin in the long-term treatment of bipolar disorder:
  Time to recurrence of any mood episodes;
  Number of recurrences of any mood episodes during the trial period;
  Number of recurrences of manic/mixed/depressive episodes during the trial period;
  Recurrence will be defined either as (i) study withdrawal due to recurrence of any mood episode, (ii) admission to hospital (time to next admission and number of admissions during trial period) or (iii) institution of additional treatment for any mood episode and time to institution.
- Efficacy of gabapentin or pregabalin in the acute and long-term treatment of anxiety is as follows:
  Change on validated and standardised anxiety rating scales.
- Efficacy of gabapentin or pregabalin in the acute and long-term treatment of insomnia is as follows:
  Objectively measured or self-reported sleep time;
  Self-reported sleep quality;
  Sleep onset latency.

#### Secondary outcomes

1. Acceptability of gabapentin or pregabalin in the acute and long-term treatment of bipolar disorder, anxiety and insomnia is measured as follows:
  - Participants dropping out of the treatment due to any cause;
  - Participants dropping out of the treatment due to adverse events;
  - Participants dropping out of the treatment due to inefficacy.
2. Tolerability (adverse events) of gabapentin or pregabalin in the acute and long-term treatment of bipolar disorder, anxiety and insomnia is measured as follows:
  - Participants experiencing at least one troublesome side effect of any nature;
  - Participants experiencing each of the following specific side effects (BNF, 2016):
    - Amnesia
    - Anxiety
    - Convulsion
    - Depression
    - Dizziness
    - Drowsiness
    - Emotional lability
    - Euphoria
    - Insomnia
    - Leucopoenia
    - Movement disorder
    - Vertigo
    - Visual disturbance
    - Weight gain

In order not to miss any relatively rare or unexpected yet important adverse events, in the data extraction phase, we will collect all side-effect data reported in the included studies and discuss ways to summarise them post hoc.

### Search methods for identification of studies

#### Electronic searches

Searches for published studies will be undertaken in the following electronic bibliographic databases: CENTRAL, EMBASE, MEDLINE, MEDLINE In-Process and PsycINFO. The following phrase will be used: ((((‘bipolar disorder’ OR ‘cyclothymic disorder’) OR ‘sleep initiation and maintenance disorders’ OR (‘anxiety’ OR ‘anxiety disorders’ OR ‘agoraphobia’ OR ‘anxiety, separation’ OR ‘combat disorders’ OR ‘neurotic disorders’ OR ‘obsessive-compulsive disorder’ OR ‘panic disorder’ OR ‘phobic disorders’ OR ‘stress disorders, traumatic’ OR ‘stress disorders, post-traumatic’ OR ‘psychological trauma’ OR ‘stress disorders, traumatic, acute’)) AND (‘gamma-aminobutyric acid’ OR ‘pregabalin’)) combined with terms for randomised controlled trials (efficacy) OR adverse effects (tolerability))*

*Subject (MeSH) entries sourced from MEDLINE.

#### Searching other resources

##### Research registers

We will search ClinicalTrials.gov and the WHO's trials portal (ICTRP) to identify unpublished or ongoing studies. There will be no date, language or publication status restrictions to the searches.

##### Grey literature

We will conduct complementary searches of the following drug approval agencies for additional published and unpublished data: The European Medicines Agency (EU), the Food and Drug Administration (USA), the Medicines and Healthcare products Regulatory Agency (UK), the Medicines Evaluation Board (the Netherlands), the Medical Products Agency (Sweden), the Pharmaceuticals and Medical Devices Agency (Japan) and the Therapeutic Goods Administration (Australia).

##### Reference lists

We will check the reference lists of all included studies, relevant papers and previous systematic reviews for identification of additional studies that may be missed by the electronic database searches. We will undertake a cited reference search of the included studies in the Web of Science.

### Data collection and analysis

#### Selection of studies

Three review authors (AF, AA, LZA) will independently check the titles and abstracts of all of the references generated by the search strategies to decide if they meet the inclusion criteria. All references potentially eligible for inclusion certified by either of the three reviewers will be added to the preliminary list, and their full-text articles will be retrieved. The three authors will then assess all the corresponding full-text articles to see if they still meet the inclusion criteria. If the authors disagree, the final decision will be made by consensus with the involvement of another member of the team.

#### Data extraction and management

KTH, AA and LZA will independently extract data from the included studies. Any disagreement will be discussed, and decisions documented. If necessary, we will contact authors of studies for clarification and original data not included in published papers. The following data will be extracted from all studies meeting the inclusion criteria:

- Study characteristics (blinding, randomisation, sponsorship, crossover/parallel group design),
- Participant characteristics (age, sex, ethnicity, study setting, primary diagnosis according to DSM or ICD classification, comorbidity, severity, treatment history for the index episode),
- Intervention details (intervention treatment, comparator treatment, dosage, frequency of administration, route of administration, duration of therapy, cointerventions),
- Outcome measures of interest in terms of efficacy, tolerability and adverse events.

#### Assessment of risk of bias in included studies

Three authors (AF, AA, LZA) will independently assess trial quality using the Cochrane risk of bias tool.^58^ The following factors will be assessed: (1) sequence generation, (2) allocation concealment, (3) blinding, (4) incomplete outcome data, (5) selective reporting and (6) other potential sources of bias. Each item will be rated as *high*, *low* or *unclear* risk of bias, and a justification from the study report will be supplied to support the judgement as appropriate. If the authors disagree, the final decision will be made by consensus with another review author (AC).

### Measures of treatment effect

#### Dichotomous data

For dichotomous, or event-like, data, the risk ratio (RR) will be calculated with its 95% CI. For statistically significant results, we will calculate the number needed to treat for an additional beneficial outcome and the number needed to treat for an additional harmful outcome, as the inverse of the risk difference.

#### Continuous data

For continuous data, mean differences (MDs) or standardised MDs (SMDs) will be calculated with 95% CIs. MDs will be used when the same scale is used to measure an outcome; SMDs will be employed when different scales are used to measure the same outcome.

Continuous data on clinical outcomes often are not normally distributed, and skewed data will be presented descriptively. If papers report a mean and an SD, as well as an absolute minimum possible value for the outcome, we will divide the mean by the SD. If this value is <2, then we will conclude that some indication of skewness is present. If the value is <1 (ie, the SD is bigger than the mean), then skewness will almost certainly be present. If papers do not report the skewness and simply report means, SDs and sample sizes, these numbers will be used. As these data may not have been properly analysed and can be misleading, fresh analyses will be conducted with and without these studies. If the data are log-transformed for analysis, and the geometric means reported, skewness will be reduced. This is the recommended method for analysis of skewed data.^58^

#### Studies with multiple treatment groups

For a particular multiarm study, the intervention groups of relevance to a systematic review are all those that could be included in a pairwise comparison of intervention groups that, if investigated alone, would meet the criteria for inclusion of studies in the review. Each meta-analysis addresses only a single pairwise comparison, so we will first consider whether a study of each possible pairwise comparison of interventions in the study is eligible for the meta-analysis. Then, several possible approaches to including a study with multiple intervention groups could be used in a particular meta-analysis.^58^ For binary outcomes, if possible, we will combine all relevant experimental intervention groups of the study into a single experimental group, and combine all relevant control intervention groups into a single control group. For continuous outcomes, we will combine means and SDs using methods described in Cochrane Handbook.^58^

#### Dealing with missing data

Binary outcomes will be calculated on a strict intention-to-treat (ITT) basis: dropouts will be included in this analysis. When data are missing and the method of last observation carried forward (LOCF) has been used to do an ITT analysis, then the LOCF data will be used.^59^ When SDs are missing, we will present data descriptively. When SDs are not reported, we will ask authors to supply the data. When only the SE or t-statistics or the p value is reported, we will calculate SDs in accordance with Altman and Bland.^60^

#### Data synthesis

We will conduct a random-effects meta-analysis, whenever possible (ideally, we plan to perform separate meta-analyses for acute and long-term treatment in each disorder).^61^ We understand that there is no closed argument for preference for the use of fixed or random-effects models. The random-effects method incorporates an assumption that different studies are estimating different, yet related, intervention effects, and takes into account differences between studies even if there is no statistically significant heterogeneity. The disadvantage of the random-effects model, though, is that it puts added weight onto small studies, which often are the most biased ones. Depending on the direction of effect, these studies can either inflate or deflate the effect size.

#### Assessment of heterogeneity

Heterogeneity between studies will be investigated by the I^2^ statistic^62^ (I^2^≥50% will be considered indicative of significant heterogeneity) and by visual inspection of the forest plots. Given that the value of I^2^ depends on the sample size of the included studies, the magnitude and direction of effects and the strength of evidence for heterogeneity, we will use arbitrary threshold to perform a preliminary evaluation. If the I^2^ value is below 50% but the direction and magnitude of treatment effects are suggestive of important heterogeneity, we will investigate the potential sources of heterogeneity.

#### Assessment of reporting biases

Data from included studies will be entered into a funnel plot (trial effect against trial variance) for investigation of small-study effects.^63^ We plan to use the tests for funnel plot asymmetry only if at least 10 studies are included in the meta-analysis.^58^ Funnel plot asymmetry may be noted for many possible reasons, so if evidence of small-study effects are identified, all possible reasons for funnel plot asymmetry, including publication bias, will be investigated.^64^

#### Subgroup and sensitivity analyses

No subgroup analysis will be undertaken. In contrast, we will carry out the following sensitivity analyses:

- excluding trials in which gabapentin or pregabalin were used as ‘add-on’ therapy to another treatment to determine if coprescription may affect the efficacy of gabapentin or pregabalin;
- excluding trials involving patients with any combination of psychiatric comorbidity;
- excluding trials which allow rescue medications (eg, short-term use of hypnotics);
- as all data will be synthesised using a random-effects model, we will also synthesise data for the primary outcome using a fixed-effects model to evaluate whether the greater weights assigned to larger trials with greater event rates can alter the significance of the results compared with the more evenly distributed weights in the random-effects model.

#### Summary of findings table

We will use the Grades of Recommendation, Assessment, Development and Evaluation approach to assess the quality of the supporting evidence behind each estimate of treatment effect. We will use risk of bias, imprecision, inconsistency, indirectness and publication bias, to rate the overall evidence.^65^ We will present key findings of the review in a ‘summary of findings’ table. This will include a summary of the amount of data, the magnitude of the effect size and the overall quality of the evidence for the primary outcomes.

### Dissemination

We will publish findings from this systematic review in a peer-reviewed scientific journal and data set will be made freely available. The completed review will be disseminated electronically, in print and on social media, where appropriate.

This protocol has been registered on PROSPERO (CRD42016041802).
